# The Effect and Safety of App-Based Interventions for Populations With Osteoarthritis: Systematic Review and Meta-Analysis of Randomized Controlled Trials

**DOI:** 10.2196/71193

**Published:** 2025-09-22

**Authors:** He Zhang, Hongchi Wang, Cheng Zhang, Ruili Zhao, Qian Lv, Luyao Zhang, Haoran Zhang, Shiyan Yan

**Affiliations:** 1 School of Management, Beijing University of Chinese Medicine, Beijing, China; 2 School of Acupuncture-Moxibustion and Tuina, Beijing University of Chinese Medicine, 11 Beisanhuan East Road, Chaoyang District, Beijing, China, 86 010-64286491; 3 School of Preschool Education, Beijing Youth Politics College, Beijing, China

**Keywords:** osteoarthritis, mobile applications, mobile health, physical therapy, meta-analysis, app

## Abstract

**Background:**

Interventions based on apps are becoming increasingly popular for the treatment of osteoarthritis (OA), but research on the potential moderators of treatment efficacy is lacking.

**Objective:**

The aim of this study was to examine the treatment efficacy, cost-effectiveness, and safety associated with app-based interventions for populations with OA and identify the potential factors associated with better treatment outcomes.

**Methods:**

PubMed, Web of Science, Embase, and the Cochrane Library were searched from their inception to September 19, 2024, for randomized controlled trials on app-based interventions for patients with OA that report efficacy or health economic outcomes. The quality of each included study was assessed using the revised Cochrane Risk of Bias Assessment Tool (ROB 2.0). The primary outcome measure is the change in pain intensity before and after treatment. Secondary outcomes included function, quality of life, adverse events, and self-management. If *I*^2^ was >50%, a random-effects model was applied. In addition to preplanned subgroup analyses based on OA type, intervention duration, risk of bias, age, and type of app-based intervention, exploratory post hoc subgroup analyses were conducted on variables related to the population, intervention characteristics, and study design features.

**Results:**

The review includes 14 studies, comprising 12 randomized controlled trials (RCTs) and 2 health economics studies. The RCTs involved a total of 1410 participants, whose mean age ranged from 54 to 67 years. Compared with controls, app-based interventions led to a reduction in pain and improvement in physical function (standardized mean difference [SMD]=−0.36; 95% CI: −0.58 to −0.14; *P*<.001; *I*^2^=72% and SMD 0.39; 95% CI 0.16 to 0.62; *P*<.001; *I*^2^=67%; respectively), but showed no significance for quality of life and adverse events (SMD 0.23; 95% CI −0.04 to 0.50; *P*=.10; *I*^2^=68% and odds ratio [OR]=1.33; 95% CI 0.84 to 2.12; *P*=.23; *I*^2^=7%; respectively). The cost of the intervention group was lower than that of the control group. Subgroup analysis revealed a significant difference between those aged 60 years and older and those younger than 60 years (SMD −0.29; 95% CI −0.51 to −0.06 and SMD −0.84; 95% CI −1.25 to −0.43). The study also reported a high level of satisfaction and compliance rate, with all scores of the System Usability Scale exceeding 70 points, and this score is considered acceptable.

**Conclusions:**

This study showed that app-based interventions were safe and effective for patients with OA, which might provide a cost-effective option, especially in resource-limited settings. Age is a critical factor for optimizing treatment benefits, especially when considering individual needs.

## Introduction

### Background

Osteoarthritis (OA) is the most common degenerative joint disorder, affecting approximately 528 million people worldwide and affecting one or multiple diarthrodial joints [1,2]. The knee, hip, and hand are the joints most frequently affected by this disease. OA imposes substantial pain and disability on patients, which reduce their quality of life (QoL) and lead to high economic costs [3-6]. With the aging population and increasing obesity rates, the prevalence of OA is expected to continue rising globally. In addition to the health burden [7], the economic burden of OA is also anticipated to increase significantly. The prevalence and alarming economic burden highlight the importance of implementing more applicable evidence-based practices specifically targeting these conditions.

Amid the COVID-19 pandemic and the challenges posed by rapid internet development, there has been a growing demand for mobile health care, with increasing interest from patients and health care providers. The World Health Organization (WHO) and Global Observatory for eHealth (GOe) [8] define mobile health (mHealth) as using appropriate digital technologies for public health. Compared with traditional care models, mHealth offers advantages such as user flexibility, allowing patients to receive remote care without having to travel. Additionally, it provides evidence-based frontline care and low-cost patient education and also holds significant potential for promoting the sustainable development of health care services. The mHealth strategies are usually used to manage chronic diseases, including diabetes, depression, asthma, and cardiovascular diseases [9-12]. Before promoting the clinical application of mHealth technology, it is crucial to fully verify its clinical efficacy and economic benefits. Previous studies have demonstrated that digital health interventions are cost-effective [13-16]. Molina-Garcia et al have confirmed the cost-effectiveness advantage of telemedicine in the management of musculoskeletal diseases through a systematic review [13]. The study shows that remote rehabilitation is US $89.55 cheaper per person than conventional treatment. Fatoye et al evaluated the economic efficiency of 2 digital health interventions for osteoarthritis, but both studies focused on telephone-based health guidance services [14]. However, synchronous interactive remote interventions such as telephone consultations not only require medical staff to have solid communication skills but also demand a significant amount of their time and energy. In contrast, application-related interventions can reach a much broader audience; by providing systematic rehabilitation education content and disease-related information, they can effectively enhance users’ willingness to actively read and improve their understanding of disease self-management [17]. The results of a 3-arm study also confirm this advantage: compared with telephone-based interventions, mobile apps are more economically advantageous and have more significant intervention effects [18]. As far as we know, there is currently no study that has carried out a systematic review and meta-analysis on the economic benefits of app-based interventions in patients with OA, and this research gap needs to be filled urgently. In OA, the primary role of mHealth apps is to provide patients with personalized health management and treatment support through smartphone apps or other mobile devices [19]. However, systematic reviews and meta-analyses of app-based interventions in OA are still relatively insufficient, and among the limited studies available, the results regarding their effects on improving patients’ QoL are somewhat inconsistent [20,21]. A systematic review and meta-analysis published in 2022 found that mobile apps supporting therapeutic exercise or tailored physical activity programs for musculoskeletal pain conditions may help reduce pain intensity and improve physical function [22]. This review included only studies on mobile apps acting as standalone digital therapeutics. Additionally, due to the restriction on publication years, a systematic review and meta-analysis on knee or hip OA published in 2020 included 12 studies, of which only 2 involved app-based interventions, and recent randomized controlled trials (RCTs) have not yet been reviewed [23]. Currently, most studies focus on the efficacy of app-based interventions [13,20-22,24], with a lack of evidence regarding their safety and the key factors that may influence treatment efficacy. In conclusion, conducting this meta-analysis is essential for resolving the discrepancies in previous studies and providing solid evidence to guide clinical practice and the development of effective digital interventions for OA.

This review aims to consolidate existing evidence to assess the efficacy, safety, and cost-effectiveness of app-based interventions for patients with OA and to identify potential factors associated with better treatment outcomes.

### Research Questions

What is the effectiveness, safety, and cost-effectiveness of app-based interventions for patients with OA, and which patient characteristics are associated with better outcomes?

## Methods

This systematic review and meta-analysis was conducted in accordance with the PRISMA (Preferred Reporting Items for Systematic Reviews and Meta-Analyses) guidelines [25]. This study was registered with PROSPERO on June 18, 2024 (CRD42024559569).

### Search Strategy

We systematically searched electronic databases, including PubMed, Web of Science, Embase, and the Cochrane Library, covering the period from database inception to September 19, 2024. The search strategy involved combining Medical Subject Headings (MeSH) terms and free text terms related to “osteoarthritis,” “applications,” and “randomized controlled trials.” (Multimedia Appendix 1). Additionally, we manually searched the references of relevant systematic reviews and meta-analyses.

### Inclusion and Exclusion Criteria

Under the PICOS (Population, Intervention, Comparison, Outcomes, and Study type) framework—a structured, evidence-based tool widely used in clinical research and systematic reviews to clarify study scope by defining key components— the inclusion criteria for our study were as follows: (1) Population: adults aged 18 years and older with OA, confirmed by X-ray, clinical diagnosis, or self-report. (2) Intervention: app-based interventions must comply with the functional classification standards for mHealth apps, established by the National Institute for Health and Care Excellence (NICE) and the National Health Service (NHS) [26]. These classifications include the following: supporting clinical diagnosis and/or decision-making; improving clinical outcomes through behavior change and enhancing patient adherence to treatment; and acting as standalone digital therapeutics and primarily delivering disease-related education. (3) Control group: conventional care, no intervention, or other nonspecific controls. (4) Outcomes: clinical effectiveness measures, economic indicators, or safety indicators. (5) Study design: randomized controlled trial (RCT).

Exclusion criteria were as follows: (1) Research on wearable devices that record only patient information. (2) Use of WeChat, Facebook, Twitter, Tencent Meeting, Zoom, or similar platforms to conduct video or message intervention research. (3) Use of websites for intervention research. (4) Studies using only phone calls and text messages for intervention. (5) Apps designed exclusively for medical staff.

### Study Selection and Data Extraction

Two independent reviewers (LZ and QL) screened titles and abstracts using EndNote software to exclude irrelevant studies. They further assessed the full texts of the remaining articles. In case of any disagreements, a third reviewer (SY) was consulted for discussion and resolution.

Data extraction was performed independently by the two reviewers (HZ and HW) using a predesigned form. The following data were recorded: basic information of study (eg, publication year, country, and authors), participant baseline characteristics (eg, number, age, gender ratio, BMI, and education level), app-based intervention features, and health economic data.

### Assessment of Risk of Bias

The quality of each included study was assessed using the revised Cochrane Risk of Bias Assessment Tool (ROB 2.0) [27], evaluating 5 domains of bias: (1) bias arising from the randomization process; (2) bias due to deviations from intended interventions; (3) bias due to missing outcome data; (4) bias in the measurement of outcomes; and (5) bias in the selection of reported results. Two independent reviewers conducted the assessments, and any disagreements were resolved through discussion until a consensus was reached. The overall risk of bias was categorized as high, some concerns, or low. When all 5 domains are assessed as having a low risk of bias, the overall assessment is categorized as low risk of bias. If at least 1 domain is identified as having a possible risk of bias and no domain is rated as having a high risk of bias, the overall assessment is categorized as some concerns. If any domain is rated as having a high risk of bias or multiple domains are identified as having a possible risk of bias, the overall assessment is categorized as high risk of bias.

### Statistical Analyses

For continuous variables, standard mean differences (SMDs) and 95% CIs were used to estimate the effect size with Hedge’s g. For the meta-analysis of adverse events, odds ratios (ORs) and 95% CIs were calculated. Heterogeneity was assessed using the *I*^2^ statistic; *I*^2^ values were calculated for the degree of heterogeneity [28]. If *I*^2^>50%, a random-effects model was applied. Sensitivity analyses were conducted by excluding studies with a high risk of bias and those with a sample size of less than 50.

In addition to preplanned subgroup analyses based on OA type, intervention duration, risk of bias, age, and type of app-based intervention, exploratory post hoc subgroup analyses were conducted on variables related to the population, intervention characteristics, and study design features. Publication bias was assessed using funnel plots and Egger regression test [29]. A *P* value <.05 was considered statistically significant. All analyses were performed using Excel (Microsoft) and Stata MP version 14.

## Results

### Study Selection

**Figure 1.**
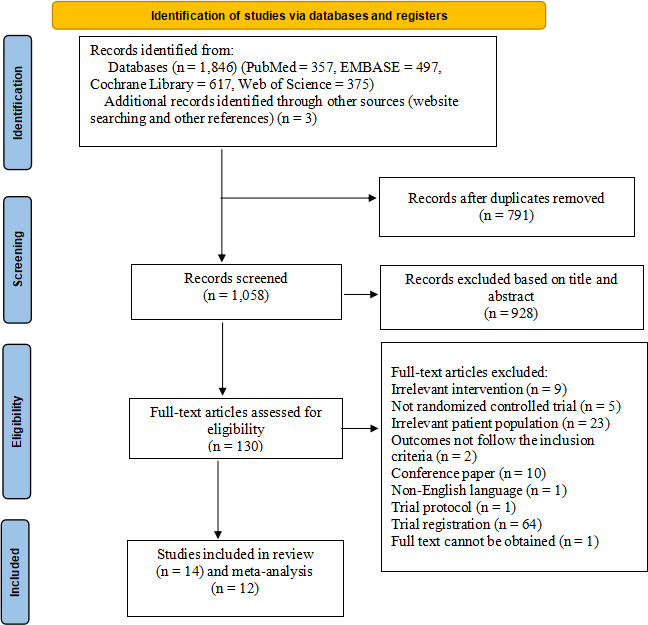
The flowchart of literature screening.

A total of 1849 potentially relevant articles were initially retrieved. After removing 791 duplicates, 1058 articles were screened based on title and abstract. Of these, 928 were excluded, and an additional 130 were excluded after a full-text review. Ultimately, 14 studies were included in the review (Figure 1).

### Study Characteristics

The study characteristics are summarized in Table 1. The review includes 14 articles published between 2017 and 2024, comprising 12 RCTs and 2 health economics studies. Of these, 10 were conducted in Western countries and 4 in Asian countries. The mean age of the participants ranged from 54 to 67 years. The RCTs involved a total of 1410 participants, with 702 (49.8%) in the intervention group and 708 (50.2%) in the control group. These studies include the following types of OA, with 8 exclusively addressing knee OA, 1 focusing on hand OA, and 3 including both knee and hip OA. Interventions based on apps lasted between 4 and 12 weeks and included features such as exercise, education, reminders, goal setting, and feedback. Four studies involved the use of wearable devices. In the control groups, 7 studies used usual care, 1 had no intervention, 1 included standard-of-care plus a wearable activity monitor, 1 used a delayed intervention, and 2 used paper-based self-guided materials.

**Table 1.** Study characteristics.

Study	Country	Conditions	Exp^a^/Con^b^	Mean age (SD) years	Interventions	Control	Duration (weeks)	Outcome(s)
			Sample size (F^c^%)					
Dieter et al [30]	Germany	Knee OA	30 (40%) /31 (58%)	62.9 (8.5)	The reflex app can be classified as a fully automated, digital health app including a training app and 2 accelerometers to monitor joint movement.	Usual care	12	Pain intensity, physical function, QoL^d^, HRQoL^e^, exercise-specific self-efficacy, isometric maximum force measurement, and postural control
Weber et al [31]	Germany	Knee OA & Hip OA	32 (65.6%) /28 (57.1%)	61.9 (7.2)	App-based Join2Move program includes exercise, physical activity, and education program.	Usual care	12	Pain intensity, physical function, usability, satisfaction, strength, ROM^f^, physical activity, self-management
Rodríguez Sánchez-Laulhé et al [32]	Spain	Hand OA	34 (73.5%) /40 (62.5%)	63.3 (8.2)	App-based care and home exercise program includes recommendations, joint protection material, general information about the disease, videos with demonstrations, and “follow-up diary.”	Usual care	12	Pain intensity, physical function, morning stiffness, and strength
Lee et al [33]	Korea	Knee OA	15 (100%)/16 (100%)	67.0 (4.4)	Exercises can be performed at home via the app exact movement and repetitions plus a remote rehabilitation medical device exoRehab.	Without any intervention	8	Pain intensity and strength
Thiengwittayaporn et al [34]	Thailand	Knee OA	42 (85.7%) /40 (62.5%)	62.6 (8.3)	“Love your knee” app modules for education and assessment for the stage of disease.	Handout exercise guidance	4	Pain intensity, physical function, ability, ROM^f^, symptoms, sports, recreation activities, and QoL^d^
Alasfour and Almarwani [35]	Saudi Arabia	Knee OA	20 (100%) /20 (100%)	54.4 (4.3)	The “My Dear Knee” app provides a guide for exercise performance, alerts, and a monitoring system.	Paper group	6	Pain intensity, physical function, and adherence
Chitkar et al [36]	Iran	Knee OA	31 (100%) / 29 (100%)	58.3 (6.9)	Patients received all the educational content through the mobile app.	Routine cares	8	Pain intensity, physical function, stiffness, and HRQoL^e^
Gohir et al [37]	UK	Knee OA	48 (70.8%)/57 (64.9%)	66.7 (9.2)	iOS (Apple) or Google Play (Alphabet) app provided the intervention group with daily exercises and informative texts.	Usual care	6	Pain intensity, physical function, stiffness, and MSK-HQ^g^
Pelle et al [4]	Netherlands	Knee OA & Hip OA	214 (68.7%) /213 (74.6%)	62.1 (7.4)	Dr. Bart app includes education, physical activity, vitality, and nutrition.	Usual care	12	Pain intensity, physical function, number of secondary health care consultations, symptoms, HRQoL^e^, and self-management
Li et al [38]	Canada	Knee OA	26 (88.5%)/25 (76.0%)	64.9 (8.4)	FitViz is a new Fitbit-compatible web-based app, participants could view their physical activity goal attainment on it plus the use of a Fitbit Flex-2 wristband.	Delay group	12	Pain intensity, physical function, mean daily MVPA^h^ time, symptoms, ADL^i^, sports/recreation, QoL^d^, self-management, and motivation
Kloek et al [39]	Netherlands	Knee OA & Hip OA	109 (67.9%) /99(67.7%)	63.1(8.7)	e-Exercise is a combination of about 5 face-to-face sessions with a physical therapist and an online application focusing on exercises and information.	Usual physical therapy	12	Pain intensity, physical activity, symptoms, QoL^d^, self-perceived effect, self-efficacy, and tiredness
Skrepnik et al [40]	USA	Knee OA	107 (55.1%)/104 (45.2%)	62.6 (9.4)	The OA GO app provided motivational messages and requested that the patient enter pain and mood data on a once-daily basis plus unblinded wearable activity monitor.	Standard of care plus wearable activity monitor	12	Pain intensity, adherence, mobility, QoL^d^, safety, and tolerability
Pelle et al [41]	Netherlands	Knee OA & Hip OA	214 (68.7%) /213 (74.6%)	62.1 (7.4)	Dr. Bart app includes education, physical activity, vitality, and nutrition.	Usual care	12	Pain intensity, physical function, number of secondary health care consultations, symptoms, HRQoL^e^, self-management, costs, and QALYs^j^
Kloek et al [42]	Netherlands	Knee OA & Hip OA	109(67.9%) / 99 (67.7%)	63.1 (8.7)	e-Exercise is a combination of about 5 face-to-face sessions with a physical therapist and an online app focusing on exercises, and information.	Usual physical therapy	12	Physical function, cost, physical activity, QALYs^j^

a Exp: experimental group.

b Con: control group.

c F: female.

d QoL: quality of life.

e HRQoL: health-related quality of life.

f ROM: range of motion.

g MSK-HQ: Musculoskeletal Health Questionnaire.

h MVPA: moderate-to-vigorous physical activity.

i ADL: activities of daily living.

j QALYS: quality-adjusted life years.

### Risk of Bias Assessment

The results of the ROB2 assessment are shown in Multimedia Appendix 2. Six studies were considered to have a high risk of bias, 2 were considered to have some concerns, and 4 were considered to have low risk of bias. Overall, the risk of bias was high due to a significant number of participant dropouts, baseline differences between groups, and failure to adhere to published protocols.

### Pain

**Figure 2.**
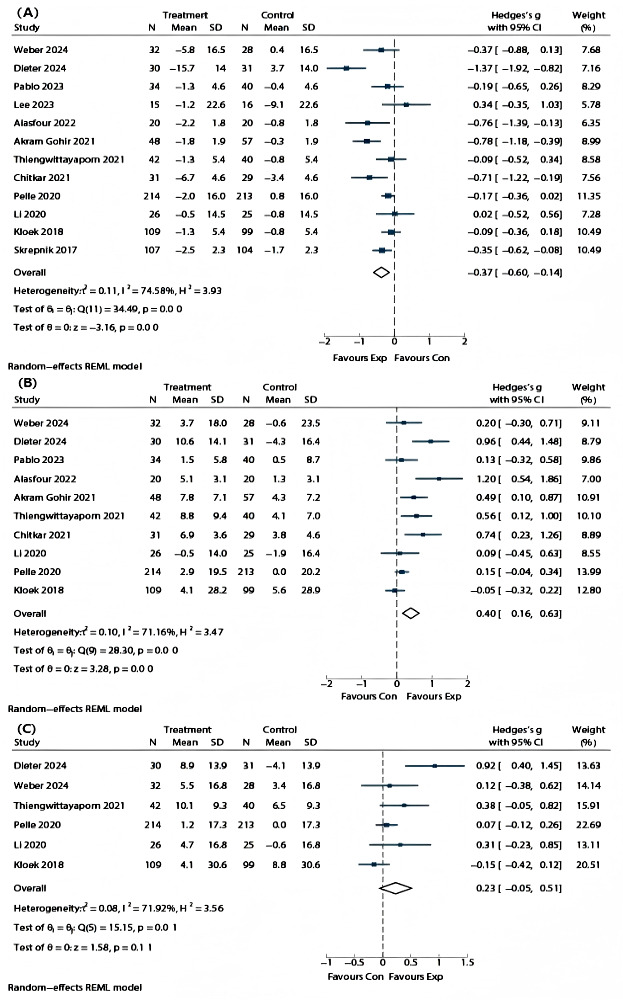
Results of meta-analyses [4,32-42] (A) pain; (B) function; and (C) QoL. Exp: experimental group; Con: control group; QoL: quality of life.

All 12 RCTs included pain intensity. Pain intensity was measured using 5 different scales: 6 studies used the KOOS/HOOS scale [4,30,31,34,38,39], 3 used the NRS scale [35,37,40], 1 used the AUSCAN scale [32], 1 used the VAS scale [31], and 1 used the WOMAC scale [36]. For patients with OA, the pooled effect of app-based interventions in reducing pain was significant (SMD −0.36; 95% CI −0.58 to −0.14; *P*<.001; *I*²=72%) (Figure 2 [4,32-42]).

**Figure 3.**
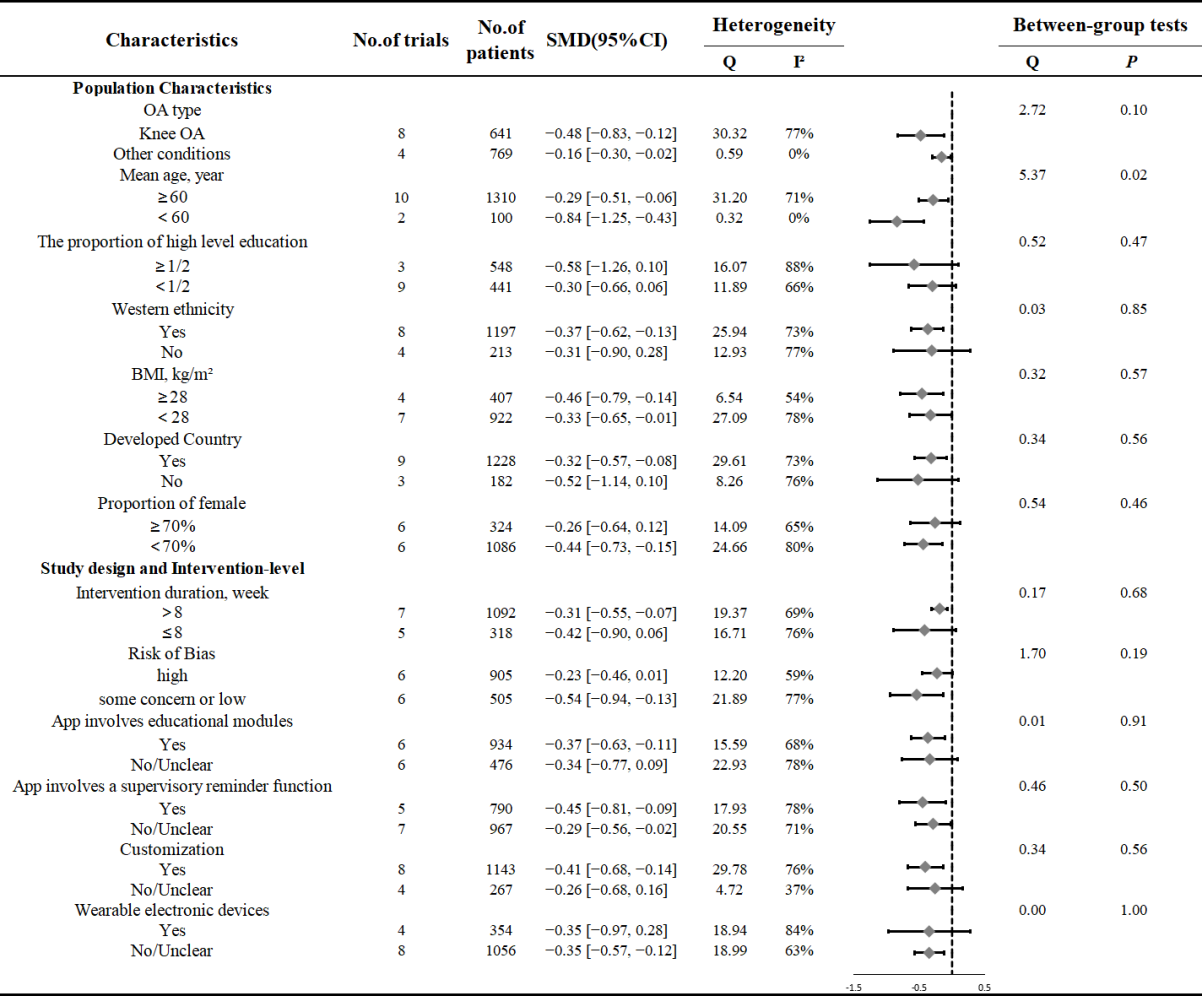
Subgroup analyses. OA: osteoarthritis; BMI: body mass index.

In subgroup analysis, regarding population characteristics, there were no significant differences between only knee OA group and other conditions group (SMD −0.48; 95% CI −0.83 to −0.12 vs SMD −0.16; 95% CI −0.30 to −0.02; *Q*=2.72; *P*=.1). However, there was a significant difference in pain reduction effects based on age, with notable differences between the 60 years and older age group and younger than 60 years age group (SMD −0.29; 95% CI −0.51 to −0.06 vs SMD −0.84; 95% CI −1.25 to −0.43; *Q*=5.37; *P*=.02). Intervention effects were not significant in the group with a higher proportion of individuals with higher education (SMD −0.58; 95% CI −1.26 to 0.10) and the group with a lower proportion of higher education (SMD −0.30; 95% CI −0.66 to 0.06). The difference in effects between these two education level groups did not reach statistical significance (*Q*=0.52; *P*=.47). Similarly, Western ethnicity, developed countries, and BMI also showed no significant group difference (Figure 3 and Multimedia Appendix 3).

Interventions with a duration of >8 weeks showed significant effects, while those with a duration of ≤8 weeks did not (SMD −0.31; 95% CI −0.55 to −0.07 vs SMD −0.42; 95% CI −0.90 to 0.06). The presence of educational modules in the app was associated with significant intervention effects, whereas the absence of educational modules was not (SMD −0.37; 95% CI −0.63 to −0.11 vs SMD −0.34; 95% CI −0.77 to 0.09). Similarly, interventions with customized app content yielded significant effects (SMD −0.35; 95% CI −0.97 to −0.28), and significant effects were also observed in studies without wearable devices (SMD −0.35; 95% CI −0.57 to −0.12). However, the differences in effectiveness across these factors did not reach statistical significance (Figure 3).

### Function

Ten studies included physical functioning as an outcome, measured using 4 different scales: 6 studies used the HOOS/KOOS scale [4,30,31,38,39], 3 used the WOMAC scale [35-37], and 1 used the AUSCAN scale [32]. The pooled effect showed that the intervention group improved physical functioning more than the control group (SMD 0.39; 95% CI 0.16 to 0.62; *P*<.001; *I*²=67%) (Figure 2 [4,32-42]).

### Safety

**Figure 4.**
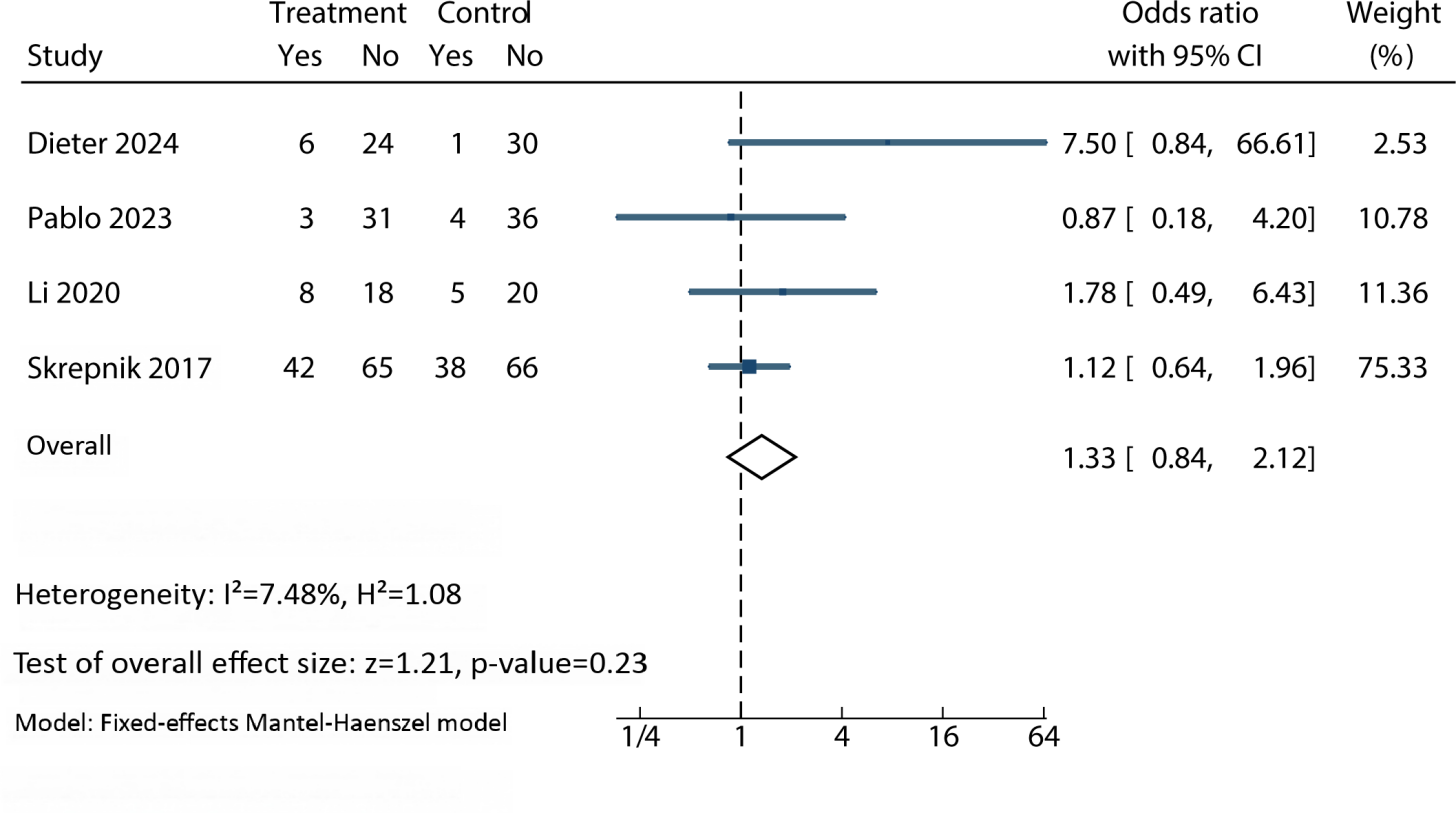
Forest plot for the efficacy of an app-based intervention on safety [30,32,38,40].

Of the 12 studies included in this meta-analysis, 2 (16.7%) explicitly reported that no adverse events had occurred [31,37], while 6 (50%) made no mention of adverse events [4,33-36,39]. Adverse events were reported in 4 studies [30,32,38,40], totaling 107 events. There were 59 adverse events among 197 participants allocated to the app intervention group. For participants allocated to usual care (n=200), 48 adverse events were reported. Of the 59 reported events, the most commonly reported adverse events among patients were muscle pain, arthralgia, upper respiratory tract infection, and falling while being physically active. There was no significant difference between groups (OR=1.33; 95% CI 0.84 to 2.12; *P*=.23; *I*²=7%) (Figure 4 [30,32,38,40]).

### Other Outcomes

#### QoL and Self-Management

**Figure 5.**
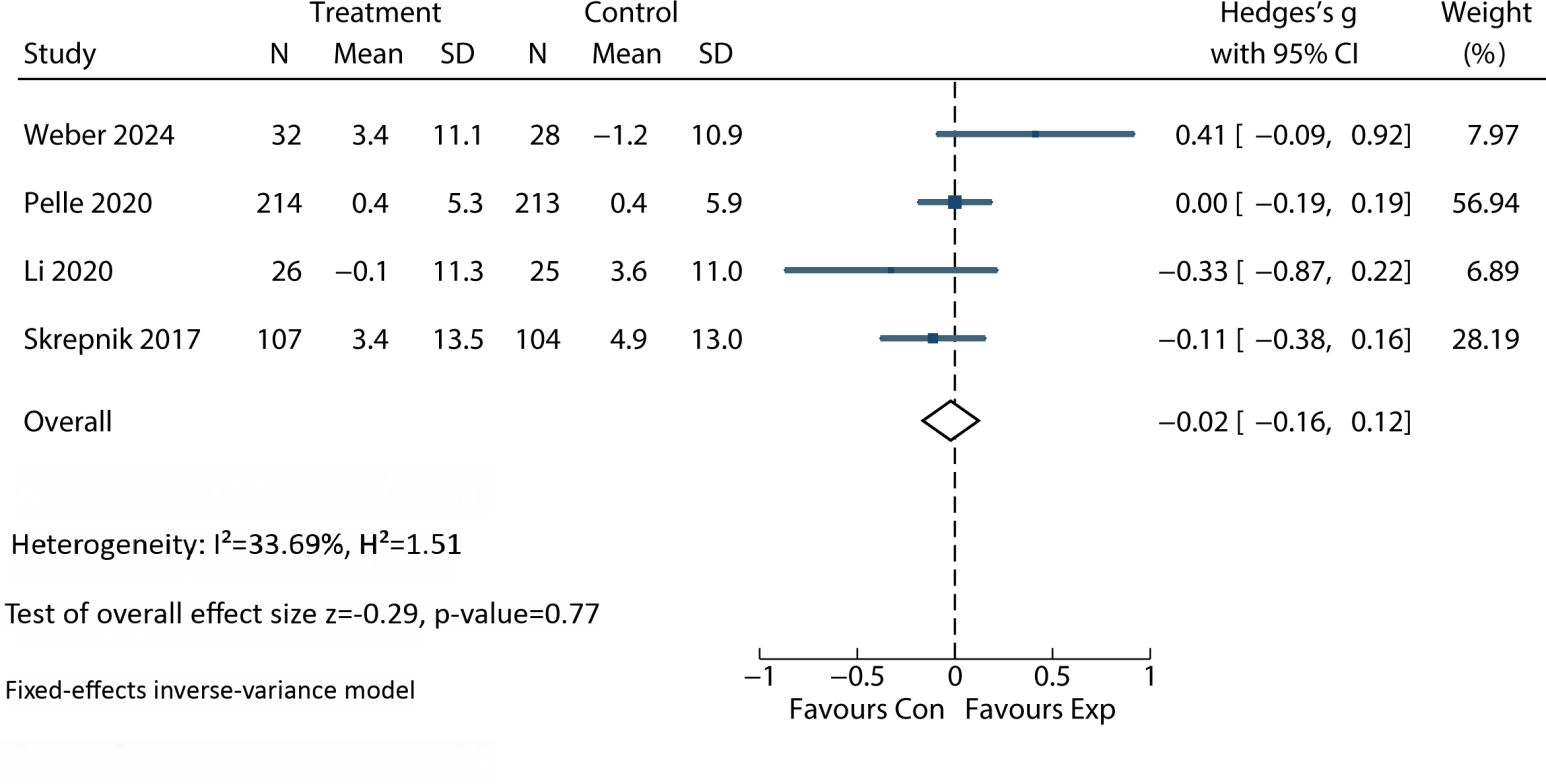
Forest plot for the efficacy of an app-based intervention on self-management [31,32,38,40].

Six studies assessed QoL as an outcome [4,30,31,34,38,39], revealing no significant difference between the intervention and control groups (SMD 0.12; 95% CI −0.01 to 0.25; *P*=.08; *I*²=68%)(Figure 2 [4,32-42] ). Additionally, 4 studies evaluated self-management [4,31,38,40], finding no significant difference between groups (SMD −0.02; 95% CI: −0.16 to 0.12; *P*=.77; *I*²=33.69%) (Figure 5 [31,32,38,40]).

#### Health Economics of App-Based Intervention

Two studies included app-based intervention cost analyses [41,42]. One study reported no significant cost difference between the intervention and control groups (MD −1371.00; 95% CI −451.00 to 1240.00) [41]. Another study performed cost-effectiveness and cost-utility analyses [42], revealing no significant difference in quality-adjusted life years between groups (MD 0.00; 95% CI −0.00 to 0.01) and a cost saving of €22 (95% CI −36.00 to −3.00).

#### Adherence, Satisfaction, and Usability

Five studies reported on compliance, which was defined as the proportion of exercise sessions completed relative to the total number of scheduled sessions, or the completion of specified modules or requirements within the app [4,30,35,38-40]. Two studies reported on satisfaction, with one using the ZUF-8 scale (0‐32) and the other using a percentage-based reporting method [31,40]. Three studies used the System Usability Scale (SUS) (0‐100) to assess usability.

### Sensitivity Analyses

We excluded RCTs with a high risk of bias and those with a sample size smaller than 50. The sensitivity analysis for pain intensity results robustly supports the intervention group (SMD −0.54; 95% CI −0.94 to −0.13; *P*<.001 and SMD −0.38; 95% CI −0.61 to −0.16; *P*<.001; respectively) (MultimediaAppendices 4  5).

### Assessment of Publication Bias

The funnel plot and Egger test (*P*=.29) indicate no significant publication bias for pain intensity (Multimedia Appendix 6).

## Discussion

### Principal Findings and Comparison With Previous Works

This systematic review and meta-analysis evaluated the effectiveness, safety, and economics of app-based interventions for patients with osteoarthritis—findings that are crucial for the development and promotion of app-based OA intervention measures. Our results show that exercise and care delivered via app-based interventions provide greater efficacy and benefits compared to control interventions, and age is a key factor in optimizing treatment outcomes.

To our knowledge, this systematic review and meta-analysis is the first comprehensive evaluation of app-based interventions for patients with OA, assessing their efficacy, safety, and economic impact, and it is also the most comprehensive study to explore the patient characteristics and research intervention factors associated with better outcomes. Overall, this review shows that app-based interventions result in significant improvements in pain intensity and physical function compared with interventions without app support. However, no significant advantages were shown in QoL and self-management. Furthermore, we did not find an increased risk of adverse events when app intervention was compared with other treatments.

Our meta-analysis results show that app-based interventions can significantly reduce pain. This finding is consistent with the results of a systematic review and meta-analysis focusing on musculoskeletal diseases within a narrower range of mHealth interventions [22], as well as with a systematic review and meta-analysis on a more extensive digital intervention for patients with OA [43].

Our study demonstrated that age of participants was a significant factor influencing the effectiveness of app-based interventions. We observed notable reductions in pain intensity for participants across age groups, especially in the subgroup of those younger than 60 years. This result is consistent with the meta-analysis by Goh et al [44]. This might be related to the prevalence of age-related comorbidities in older patients or differences in app intervention acceptance and preferences. Lim et al’s study on consumer preferences for mHealth apps found that younger individuals (18‐54 y) are more inclined to use these apps [45]. However, the studies included in this review primarily involved participants aged between their 50s and 60s. This suggests that the findings may not be generalizable to younger or older populations, highlighting the need for further research to evaluate the efficacy of app-based interventions across a broader age range. Additionally, it was noted that higher-income individuals were more likely to use apps, and in the subgroup analysis of whether the study was conducted in a developed country, better outcomes were observed in developed countries, although the differences were not statistically significant. This finding appears to support the conclusion. Then, it is noteworthy that studies on apps with supervisory reminder features, and those with unclear/no reminder features did not show a difference in the effectiveness of app-based interventions. Intervention groups demonstrated better pain relief compared with the control group, indicating that app-based interventions have a positive effect on pain reduction overall, regardless of the presence of reminder features. However, this might be due to some studies not specifying whether the app had reminder features, leading to a substantial amount of error by grouping them as having unclear/no reminders. While differences in effectiveness were observed regarding whether the app included an educational component and whether it was customized, these differences were not statistically significant. This lack of significance might be attributed to small sample sizes and considerable heterogeneity among studies. Interventions using apps with educational components and customization showed better outcomes, though not statistically significant, suggesting potential advantages in practical applications [46]. This warrants further investigation in future research.

As for other results, the meta-analysis results indicate that app-based interventions offer benefits in improving physical function. This is consistent with previous research [25,46]. This consistency may be related to the core functions of app-based interventions—most intervention-oriented apps help patients develop regular rehabilitation habits through standardized rehabilitation training guidance, such as joint range of motion exercises and muscle-strengthening movements, or phased goal management, thereby improving physical function. This also indirectly confirms the stability of apps as rehabilitation auxiliary tools in improving physical function.

The results of this study showed no significant difference in the number of adverse events between the app intervention group and the control group, which may be attributed to the fact that only a few of the included studies reported adverse events. The reported adverse events mainly focused on musculoskeletal pain and falls after exercise. However, it is noteworthy that the greatest potential risk of using digital health technologies lies in the privacy and security of patients’ personal data. Regrettably, none of the studies included in this research reported on this aspect. In view of this, future studies should attach great importance to the protection of patients’ data privacy and incorporate it into the monitoring and evaluation system of adverse events, so as to comprehensively assess the safety and reliability of digital health interventions.

Only 2 studies assessed the cost differences between app-based interventions and control groups. One study showed cost-saving benefits [41], while the other did not [42]. However, prior economic evaluations of digital health interventions for musculoskeletal diseases have found that these interventions are cost-effective compared with control groups [14]. Similar positive cost-effectiveness has also been observed for people with mental disorders and those with type 2 diabetes mellitus [15,16,47,48]. Therefore, researchers should place greater emphasis on the health economic outcomes of app-based interventions in future studies evaluating their effects on OA.

However, there was no significant improvement in QoL or self-management abilities compared with the control group. This might be due to the multidimensional nature of QoL, which is not solely influenced by disease-specific factors [49]. Only 3 studies reported self-management outcomes, with significant variability. Previous research has indicated that being an engaged and active participant in one’s own care is associated with better health outcomes [50]. The NICE guidance committee suggests that patients should actively participate in and manage their care [51]. Future research could further explore how app-based interventions can effectively enhance patients’ self-management skills and health engagement, with a focus on developing personalized apps and their potential role in long-term health improvement.

Additionally, high satisfaction and adherence rates were reported, aligning with previous findings in OA and other conditions [52]. Particularly in terms of usability, all 3 studies reported SUS scores exceeding 70 points, with scores above this threshold considered acceptable. These results suggest the potential of app-based interventions as relatively low-cost alternatives to traditional treatments in settings with limited resources or funding.

The mHealth apps in this study provide exercise and physical activity support, as well as personalized and tailored health information and education. The exercise interventions in this study include aerobic exercise, muscle strength training, and joint range-of-motion training. Previous systematic reviews and meta-analyses have confirmed that exercise therapy can significantly reduce pain and improve joint function, and the conclusions of this study are consistent with these findings [53,54]. As a flexible and efficient rehabilitation solution, mobile or remote exercise interventions based on the app can completely break through the time and location constraints of traditional treatment—patients do not need to make frequent trips to and from the hospital and can arrange rehabilitation training flexibly according to their own schedules. In terms of practical effects, it can not only significantly improve the overall efficiency of exercise therapy but also greatly reduce patients’ waiting time. Economic evaluations have found that physical therapy supported by remote apps can effectively reduce costs, and its therapeutic effect is equivalent to that of the traditional offline model [55].

The ROB assessment highlights that the lack of blinding among participants and excessive missing data are significant sources of bias in the studies. Implementing blinding is relatively challenging due to the nature of app-based interventions. Additionally, the autonomy associated with home exercise and app usage may result in many participants being unable to adhere to the interventions. The broad scope of OA, coupled with the inclusion of various types of app-based interventions and control groups in this study, has led to considerable heterogeneity within the population. These factors may affect the reliability and applicability of the study findings.

### Limitations

This study has several limitations. First, the number of included studies was relatively small, which was due to the stringent inclusion criteria applied. Second, while only 2 studies included economic evaluations, which necessitates caution in interpreting the economic findings, existing international research has established that app-based interventions are cost-effective in the management of other diseases [13,56]. It is recommended that prospective studies be conducted in the future to rigorously evaluate its health economics. Third, only English language studies were included. Focusing solely on English language publications may result in the omission of relevant studies conducted in other languages, potentially leading to an incomplete understanding of the effectiveness of app-based interventions across different cultural contexts and health care systems. Consequently, the generalizability of the conclusions may be constrained. Fourth, in an age-based subgroup analysis, it should be noted that only 2 studies were included in this subgroup with a small sample size. The age range of the population included in this paper is relatively narrow. Therefore, future studies should enroll participants from different age groups and explore the associations between age and potential moderating factors of treatment efficacy. Additionally, the results of such small sample subgroups in the subgroup analysis should also be interpreted with caution. Finally, the interventions evaluated had relatively short duration, leaving their long-term efficacy unclear.

### Conclusions

These systematic review and meta-analysis confirm that app-based interventions are an effective and safe treatment option for patients with OA. Our study also underscores the importance of considering individual characteristics, particularly age, as a key factor influencing the effectiveness of app-based interventions. Younger patients tended to show greater improvements, which suggests that age-tailored approaches could enhance the efficacy of these digital solutions. These findings are expected to serve as a reference for developers and researchers in the rapidly evolving field of mHealth, offering guidance on the design, promotion, and implementation of app-based interventions for OA.

## Supplementary material

10.2196/71193 Multimedia Appendix 1 Details of the literature search and the number of citations found.

10.2196/71193 Multimedia Appendix 2 Risk of bias of included studies.

10.2196/71193 Multimedia Appendix 3 Characteristics of subgroup.

10.2196/71193 Multimedia Appendix 4 Results of sensitivity analyses: excluded RCTs with a high risk of bias.

10.2196/71193 Multimedia Appendix 5 Results of sensitivity analyses: excluded RCTs with a sample size smaller than 50.

10.2196/71193 Multimedia Appendix 6 Funnel plot assessing publication bias.

10.2196/71193 Checklist 1 PRISMA checklist.

## Abbreviation

GOe: Global Observatory for eHealth
MeSH: medical subject headings
mHealth: mobile health
NHS: National Health Service
NICE: National Institute for Health and Care Excellence
OA: osteoarthritis
OR: odds ratio
PICOS: Population, Intervention, Comparison, Outcomes, and Study type
PRISMA: Preferred Reporting Items for Systematic Reviews and Meta-analyses
QoL: quality of life
RCT: randomized controlled trial
ROB: risk of bias
SMD: standardized mean difference
WHO: World Health Organization
