# Supplementary material for: The Effect and Safety of App-Based Interventions for Populations With Osteoarthritis: Systematic Review and Meta-Analysis of Randomized Controlled Trials
Source: JMIR Mhealth Uhealth. 2025 Sep 22;13:e71193. doi: 10.2196/71193 (PMC12454192; doi:10.2196/71193)
Supplement: Multimedia Appendix 1 [file mhealth-v13-e71193-s001.docx]

**Multimedia Appendix 1**

**Methods 1. Details of the literature search and the number of citations found.**

**Pubmed:**

| **Search number** | **Query** | **Results** |
| --- | --- | --- |
| #10 | ((#3) AND (#6)) AND (#9) | 613 |
| #9 | (#7) OR (#8) | 499,055 |
| #8 | (((((((((((((((((((mobile application[Title/Abstract]) OR (smartphone application[Title/Abstract])) OR (mobile[Title/Abstract])) OR (smartphone[Title/Abstract])) OR (app[Title/Abstract])) OR (mobile app[Title/Abstract])) OR (mobile health[Title/Abstract])) OR (mhealth[Title/Abstract])) OR (m-health[Title/Abstract])) OR (app-based[Title/Abstract])) OR (mobile-based[Title/Abstract])) OR (phone-based[Title/Abstract])) OR (smartphone-based[Title/Abstract])) OR (e-health[Title/Abstract])) OR (ehealth[Title/Abstract])) OR (Telehealth[Title/Abstract])) OR (Telecare[Title/Abstract])) OR (cell phone[Title/Abstract])) OR (ipad[Title/Abstract])) OR (computer[Title/Abstract]) | 471,938 |
| #7 | ((Telemedicine[MeSH Terms]) OR (Mobile Applications[MeSH Terms])) OR (Telerehabilitation[MeSH Terms]) | 56,233 |
| #6 | (#4) OR (#5) | 156,061 |
| #5 | ((((((((((((((Osteoarthr*[Title/Abstract]) OR (Degenerative Arthritis[Title/Abstract])) OR (Arthrosis[Title/Abstract])) OR (Wrist Osteoarthritis[Title/Abstract])) OR (Hip Osteoarthritis[Title/Abstract])) OR (Knee Osteoarthritis[Title/Abstract])) OR (Hand Osteoarthritis[Title/Abstract])) OR (Osteoarthritis Of Hip[Title/Abstract])) OR (Osteoarthritis of Knee[Title/Abstract])) OR (Osteoarthritis Of Hips[Title/Abstract])) OR (Osteoarthritis of the Hip[Title/Abstract])) OR (Knee Osteoarthritides[Title/Abstract])) OR (Osteoarthritis of the Knee[Title/Abstract])) OR (Lumbar Osteoarthritis[Title/Abstract])) OR (Osteoarthritis of hand[Title/Abstract]) | 106,111 |
| #4 | (((Osteoarthritis[MeSH Terms]) OR (Osteoarthritis, Spine[MeSH Terms])) OR (Osteoarthritis, Knee[MeSH Terms])) OR (Osteoarthritis, Hip[MeSH Terms]) | 110,149 |
| #3 | (#1) OR (#2) | 1,741,096 |
| #2 | ((((((Clinical Trial[Title/Abstract]) OR (Controlled Clinical Trial[Title/Abstract])) OR (Randomized Controlled Trial[Title/Abstract])) OR (randomized[Title/Abstract])) OR (randomised[Title/Abstract])) OR (randomly[Title/Abstract])) OR (trial[Title/Abstract]) | 1,532,197 |
| #1 | ((Controlled Clinical Trials as Topic[MeSH Terms]) OR (Clinical Trials as Topic[MeSH Terms])) OR (Randomized Controlled Trials as Topic[MeSH Terms]) | 388,591 |

**the Cochrane Library：**

| **Search number** | **Query** | **Results** |
| --- | --- | --- |
| #1 | MeSH descriptor: [Osteoarthritis] explode all trees | 10785 |
| #2 | MeSH descriptor: [Osteoarthritis, Hip] explode all trees | 1351 |
| #3 | MeSH descriptor: [Osteoarthritis, Knee] explode all trees | 6708 |
| #4 | MeSH descriptor: [Osteoarthritis, Spine] explode all trees | 16 |
| #5 | (Osteoarthr*):ti,ab,kw OR (Degenerative Arthritis):ti,ab,kw OR (Arthrosis):ti,ab,kw OR (Wrist Osteoarthritis):ti,ab,kw OR (Hip Osteoarthritis):ti,ab,kw | 24660 |
| #6 | (Knee Osteoarthritis):ti,ab,kw OR (Hand Osteoarthritis):ti,ab,kw OR (Osteoarthritis Of Hip):ti,ab,kw OR (Osteoarthritis of Knee):ti,ab,kw OR (Osteoarthritis Of Hips):ti,ab,kw | 20151 |
| #7 | (Osteoarthritis of the Hip):ti,ab,kw OR (Knee Osteoarthritides):ti,ab,kw OR (Osteoarthritis of the Knee):ti,ab,kw OR (Lumbar Osteoarthritis):ti,ab,kw OR (Osteoarthritis of hand):ti,ab,kw | 19434 |
| #8 | #1OR#2OR#3OR#4OR#5OR#6OR#7 | 24660 |
| #9 | MeSH descriptor: [Telemedicine] explode all trees | 4786 |
| #10 | MeSH descriptor: [Mobile Applications] explode all trees | 1927 |
| #11 | MeSH descriptor: [Telerehabilitation] explode all trees | 333 |
| #12 | (mobile application):ti,ab,kw OR (smartphone application):ti,ab,kw OR (mobile):ti,ab,kw OR (smartphone):ti,ab,kw OR (app):ti,ab,kw | 24919 |
| #13 | (mobile app):ti,ab,kw OR (mobile health):ti,ab,kw OR (mhealth):ti,ab,kw OR (m-health):ti,ab,kw OR (app-based):ti,ab,kw | 12474 |
| #14 | (mobile-based):ti,ab,kw OR (phone-based):ti,ab,kw OR (smartphone-based):ti,ab,kw OR (e-health):ti,ab,kw OR (ehealth):ti,ab,kw | 4102 |
| #15 | (Telehealth):ti,ab,kw OR (Telecare):ti,ab,kw OR (cell phone):ti,ab,kw OR (ipad):ti,ab,kw OR (computer):ti,ab,kw | 59479 |
| #16 | #9OR#10OR#11OR#12OR#13OR#14OR#15 | 83783 |
| #17 | #8AND#16 | 1381 |

**EMBASE：**

| **Search number** | **Query** | **Results** |
| --- | --- | --- |
| #17 | #10 AND #11 AND #16 | 864 |
| #16 | #12 OR #13 OR #14 OR #15 | 637877 |
| #15 | 'mobile application':ab,ti OR 'smartphone application':ab,ti OR mobile:ab,ti OR smartphone:ab,ti OR app:ab,ti OR 'mobile app':ab,ti OR 'mobile health':ab,ti OR mhealth:ab,ti OR 'm health':ab,ti OR 'app based':ab,ti OR 'mobile based':ab,ti OR 'phone based':ab,ti OR 'smartphone based':ab,ti OR 'e health':ab,ti OR ehealth:ab,ti OR telecare:ab,ti OR 'cell phone':ab,ti OR ipad:ab,ti OR computer:ab,ti | 559787 |
| #14 | 'telerehabilitation'/exp OR 'telerehabilitation' | 3880 |
| #13 | 'mobile application'/exp OR 'mobile application' | 28463 |
| #12 | 'telemedicine'/exp OR 'telemedicine' | 87303 |
| #11 | #5 OR #6 OR #7 OR #8 OR #9 | 214929 |
| #10 | #1 OR #2 OR #3 OR #4 | 2441547 |
| #9 | osteoarthr*:ab,ti OR 'degenerative arthritis':ab,ti OR arthrosis:ab,ti OR 'wrist osteoarthritis':ab,ti OR 'hip osteoarthritis':ab,ti OR 'knee osteoarthritis':ab,ti OR 'hand osteoarthritis':ab,ti OR 'osteoarthritis of hip':ab,ti OR 'osteoarthritis of knee':ab,ti OR 'osteoarthritis of hips':ab,ti OR 'osteoarthritis of the hip':ab,ti OR 'knee osteoarthritides':ab,ti OR 'osteoarthritis of the knee':ab,ti OR 'lumbar osteoarthritis':ab,ti | 143974 |
| #8 | 'hip osteoarthritis'/exp OR 'hip osteoarthritis' | 15995 |
| #7 | 'knee osteoarthritis'/exp OR 'knee osteoarthritis' | 49304 |
| #6 | 'spinal osteoarthritis'/exp OR 'spinal osteoarthritis' | 164 |
| #5 | 'osteoarthritis'/exp OR 'osteoarthritis' | 205914 |
| #4 | 'clinical trial':ab,ti OR 'controlled clinical trial':ab,ti OR randomized:ab,ti OR randomly:ab,ti OR trial:ab,ti OR 'randomized controlled trial':ab,ti OR randomised:ab,ti | 2186455 |
| #3 | 'randomized controlled trial (topic)'/exp OR 'randomized controlled trial (topic)' | 269953 |
| #2 | 'controlled clinical trial (topic)'/exp OR 'controlled clinical trial (topic)' | 279352 |
| #1 | 'clinical trial (topic)'/exp OR 'clinical trial (topic)' | 456409 |

**Web of science:**

| **Search number** | **Query** | **Results** |
| --- | --- | --- |
| #4 | #3 AND #2 AND #1 | 604 |
| #3 | TS=(Telemedicine) OR TS=(Mobile Applications) OR TS= (Telerehabilitation) OR TS=(mobile application) OR TS=(smartphone application) OR TS=(mobile) OR TS=(smartphone) OR TS=(app) OR TS=(mobile app) OR TS=(mobile health) OR TS=(mhealth) OR TS=(m-health) OR TS=(app-based) OR TS=(mobile-based) OR TS=(phone-based) OR TS=(smartphone-based) OR TS=(e-health) OR TS=(ehealth) OR TS=(Telehealth) OR TS=(Telecare) OR TS=(cell phone) OR TS=(ipad) OR TS= (computer) | 771,324 |
| #2 | TS=(Osteoarthritis) OR TS= (Osteoarthritis, Spine) OR TS= (Osteoarthritis, Knee) OR TS= (Osteoarthritis, Hip) OR TS=(Osteoarthr*) OR TS= (Degenerative Arthritis) OR TS= (Arthrosis) OR TS=(Wrist Osteoarthritis) OR TS=(Hip Osteoarthritis) OR TS=(Knee Osteoarthritis) OR TS=(Hand Osteoarthritis) OR TS=(Osteoarthritis Of Hip) OR TS= (Osteoarthritis of Knee) OR TS= (Osteoarthritis Of Hips) OR TS= (Osteoarthritis of the Hip) OR TS= (Knee Osteoarthritides) OR TS= (Osteoarthritis of the Knee) OR TS=(Lumbar Osteoarthritis) OR TS=(Osteoarthritis of hand) | 108,485 |
| #1 | TS=(Controlled Clinical Trials) OR TS=(Clinical Trials) OR TS=(Randomized Controlled Trials) OR TS=(Clinical Trial) OR TS= (Controlled Clinical Trial) OR TS= (Randomized Controlled Trial) OR TS=(randomized) OR TS=(randomised) OR TS= (randomly) ORTS= (trial) | 1985,005 |
