# Supplementary figures and images for: The Effect and Safety of App-Based Interventions for Populations With Osteoarthritis: Systematic Review and Meta-Analysis of Randomized Controlled Trials
Source: JMIR Mhealth Uhealth. 2025 Sep 22;13:e71193. doi: 10.2196/71193 (PMC12454192; doi:10.2196/71193)

**Multimedia Appendix 2**


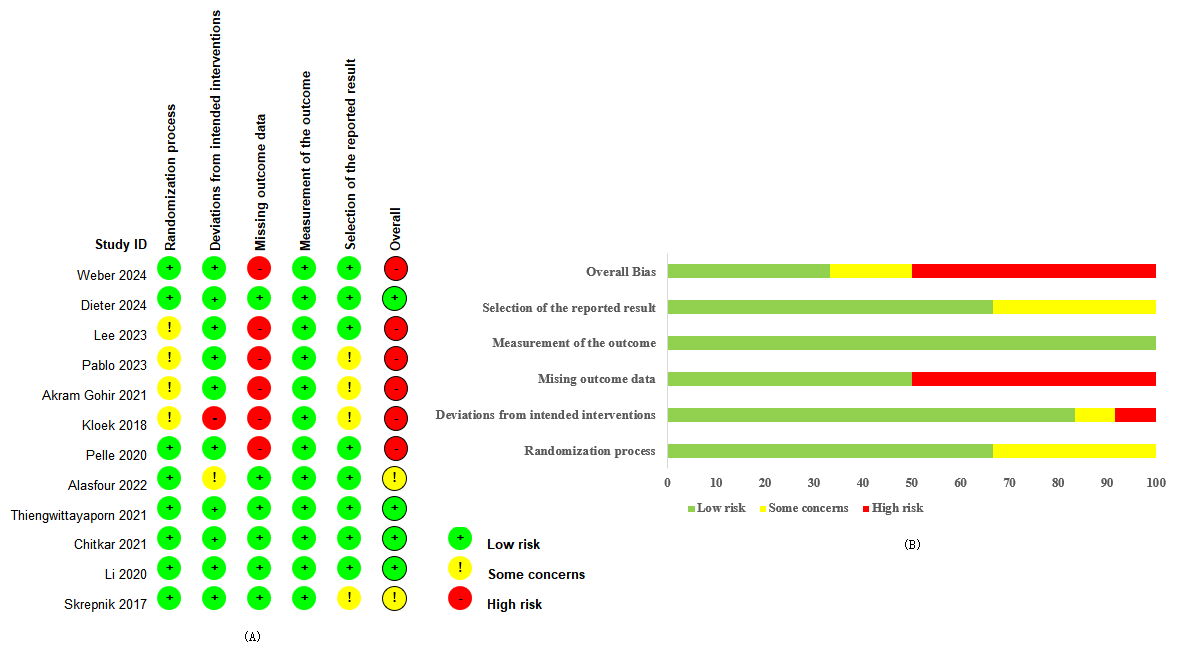


**Figure S1. Risk of bias of included studies.**

Supplement: Multimedia Appendix 2 [file mhealth-v13-e71193-s002.docx]

**Multimedia Appendix 5**

**
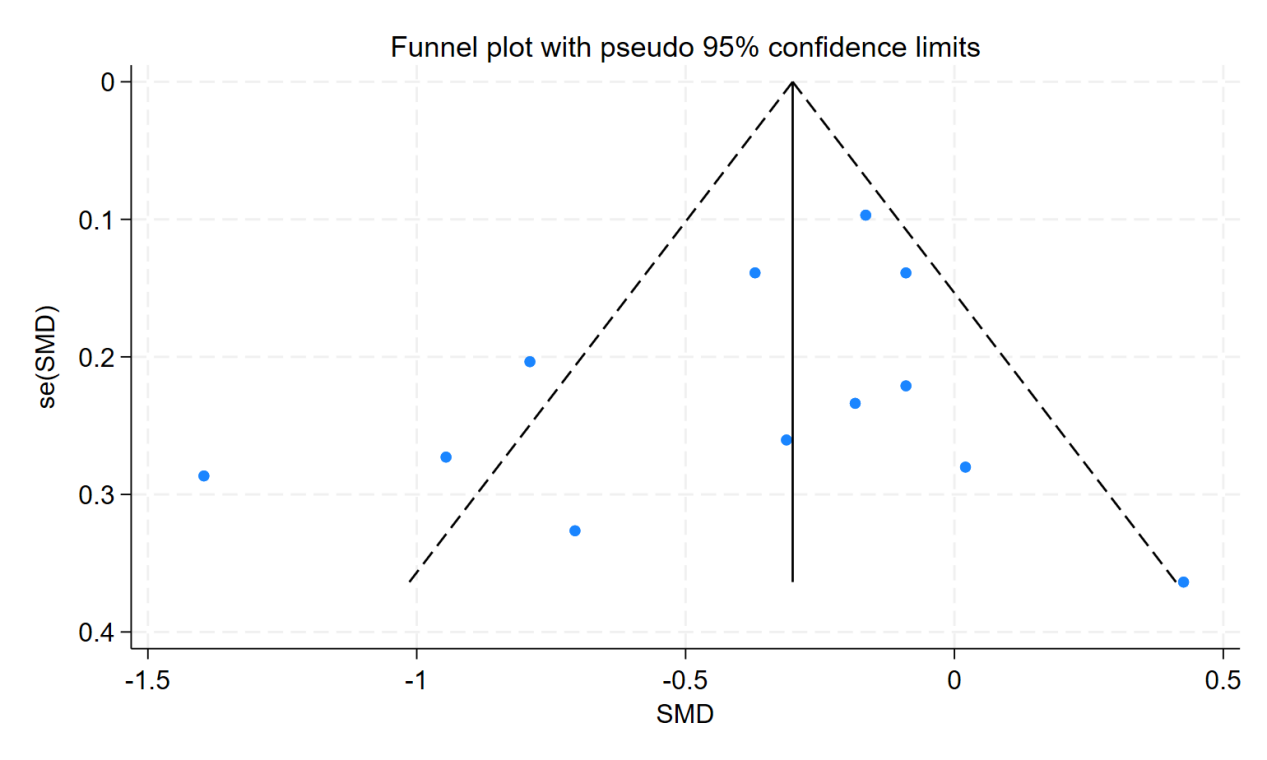
**

**Figure S4. Funnel plot assessing publication bias.**

Supplement: Multimedia Appendix 6 [file mhealth-v13-e71193-s006.docx]
