# Supplementary material for: The Effect and Safety of App-Based Interventions for Populations With Osteoarthritis: Systematic Review and Meta-Analysis of Randomized Controlled Trials
Source: JMIR Mhealth Uhealth. 2025 Sep 22;13:e71193. doi: 10.2196/71193 (PMC12454192; doi:10.2196/71193)
Supplement: Multimedia Appendix 3 [file mhealth-v13-e71193-s003.docx]

**Multimedia Appendix 6**

**Table S1. Characteristics of Subgroup.**

| High level Education | 53% | NA | NA | NA | 37% | 78.50% | 40% | 13.40% | 6.70% | NA | 49% | 53% |
| --- | --- | --- | --- | --- | --- | --- | --- | --- | --- | --- | --- | --- |
| BMI (SD), kg/m2 | 28 (6.0) | 24.72（3.2） | NA | 31.2(5.7) | 27.8(4.5) | 27.6(5.0) | NA | 26.8(4.9) | 27.31(4.0) | 29.3(3.7) | 29.4 (7.7) | 27.7(4.5) |
| Wearable device | No | Yes | No | No | No | No | No | No | No | Yes | Yes | Yes |
| App functionalities | Activity,Education,Exercise | Exercise | Exercise,Education | Exercise,Education | Activity,Education,Exercise | Education,Exercise | Exercise | Exercise | Education,Usual care | Motivation,Activity information | Activity information | Exercise |
| Customization | Yes | Yes | NA | Yes | Yes | Yes | Yes | NA | NA | Yes | NA | YES |
| Supervised | Yes | Yes | Yes | Yes | Yes | Yes | Yes | NA | NA | NA | NA | Yes |
| Duration | 12w | 8w | 12w | 6w | 12w | 6m | 6w | 4w | 6w | 12w | 12w | 12w |
| Frequency | Three times a week | Three times a week | Four times a week | NA | Three times a week | NA | Once a day | NA | NA | Everday | NA | Three times a week |
| Average age | 61.9±7.2 | 67±4.4 | 63.3±8.2 | 66.7±9.2 | 63.1±8.7 | 62.1±7.4 | 54.4±4.3 | 62.6±8.3 | 58.3±6.9 | 62.6±9.4 | 64.9±8.4 | 62.9±8.5 |
| Female proportion | 61.67% | 100% | 67.57% | 67.62% | 67.79% | 71.66% | 100% | 89.02% | 100% | 50.24% | 82.35% | 49% |
| Western ethnicity | Yes | No | Yes | Yes | Yes | Yes | No | No | No | Yes | Yes | Yes |
| Developed country | Yes | Yes | Yes | Yes | Yes | Yes | No | No | No | Yes | Yes | Yes |
| Country | German | Korea | Spain | UK | Netherlands | Netherlands | Saudi Arabia | Thailand | Iran | USA | Canada | German |
| Study | Weber 2024 | Lee 2023 | Pablo 2023 | Akram Gohir 2021 | Kloek 2018 | Pelle 2020 | Alasfour 2022 | Thiengwittayaporn 2023 | Chitkar 2021 | Skrepnik 2017 | Li 2020 | Dieter 2024 |

table. 1
