# Supplementary material for: The Effect and Safety of App-Based Interventions for Populations With Osteoarthritis: Systematic Review and Meta-Analysis of Randomized Controlled Trials
Source: JMIR Mhealth Uhealth. 2025 Sep 22;13:e71193. doi: 10.2196/71193 (PMC12454192; doi:10.2196/71193)
Supplement: Multimedia Appendix 5 [file mhealth-v13-e71193-s005.docx]

**Multimedia Appendix 4**


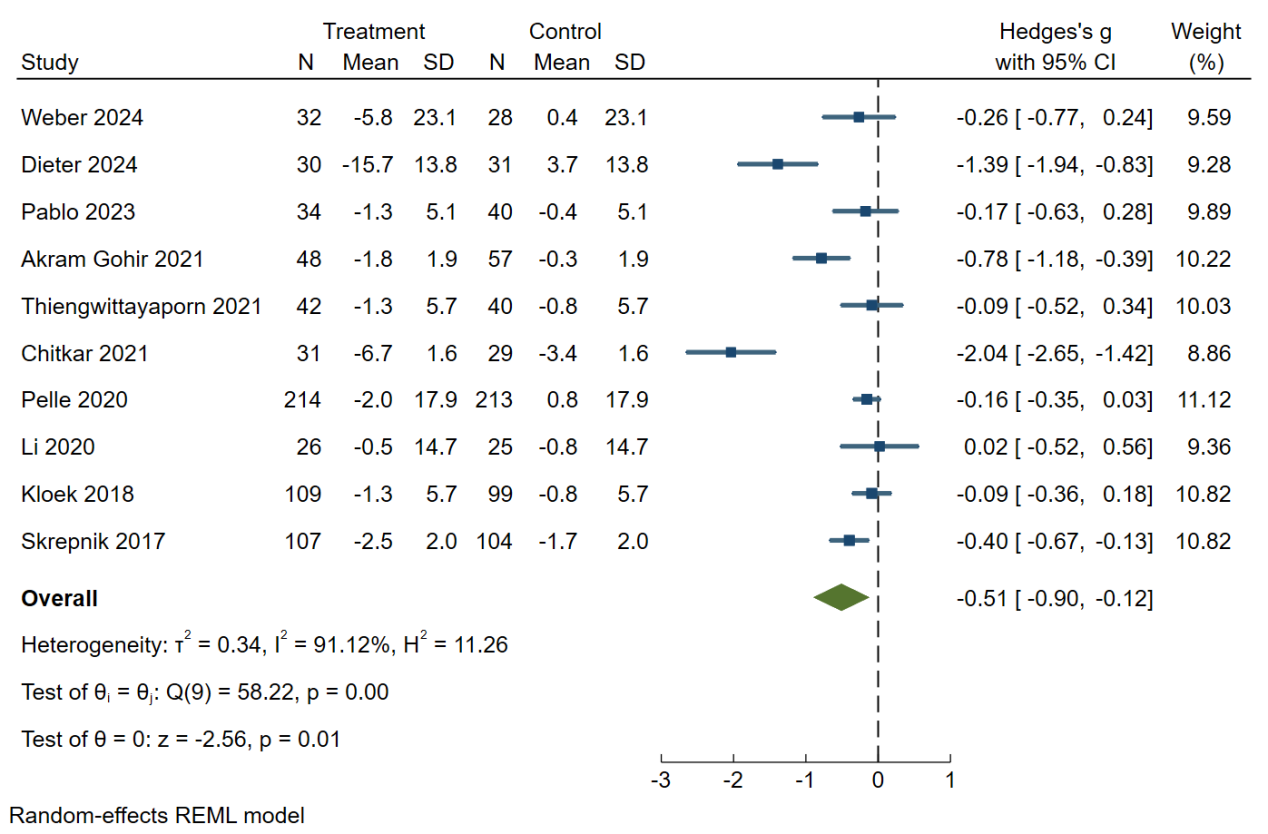


**Figure S3. Results of sensitivity analyses: excluded RCTs with a sample size smaller than 50.**
